# Cognition, white matter hyperintensities and suicide risk in late-life depression patients: an exploratory study

**DOI:** 10.1186/s12877-025-06358-x

**Published:** 2025-09-03

**Authors:** Yao-Tung Lee, Li-Kai Huang, Martha Sajatovic, Chaur-Jong Hu, Hsin-Chien Lee, Che-Yin Lin, Chin-An Wang, Shang-Ying Tsai, Yen-Kuang Lin

**Affiliations:** 1 Department of Psychiatry and Psychosomatic Medicine, Far Eastern Memorial Hospital, New Taipei City, Taiwan; 2 Department of Psychiatry, Shuang Ho Hospital, Taipei Medical University, New Taipei City, Taiwan; 3 Department of Neurology, Shuang Ho Hospital, Taipei Medical University, New Taipei City, Taiwan; 4 Dementia Center, Shuang Ho Hospital, Taipei Medical University, New Taipei city, Taiwan; 5 Department of Psychiatry and of Neurology, Neurological and Behavioral Outcomes Center, Case Western Reserve University School of Medicine, University Hospitals Cleveland Medical Center, 10524 Euclid Avenue, Cleveland, OH 44106 USA; 6 Department of Psychiatry, Taipei Medical University Hospital, Taipei Medical University, Taipei,, Taiwan; 7 Department of Psychiatry, School of Medicine, College of Medicine, Taipei Medical University, Taipei,, Taiwan; 8 Department of Anesthesiology, Shuang Ho Hospital, Taipei Medical University, New Taipei City, Taiwan; 9 Department of Anesthesiology, School of Medicine, College of Medicine, Taipei Medical University, Taipei,, Taiwan; 10 Graduate Institute of Athletics and Coaching Science / Director, International Affairs and Chinese Language Center, National Taiwan Sport University, Taoyuan,, Taiwan

**Keywords:** Magnetic resonance imaging, White matter hyperintensities, Cognition, Vascular depression, Aged, Suicide, Attempted

## Abstract

**Objective:**

Suicide among older adults represents a major public health concern and is closely associated with late-life depression (LLD). White matter hyperintensities (WMHs), frequently observed in the aging population, have been linked to both LLD and cognitive impairment. However, the role of WMH burden and cognitive dysfunction in older adults with LLD who have recently attempted suicide remains unclear. This study aims to investigate differences in WMH burden and cognitive performance across three groups: LLD patients with a recent suicide attempt (recent suicide attempt group, RSA), non-suicidal LLD patients (non-suicidal group, NS), and healthy older adults (healthy comparison group, HC). We further examine whether the relationship between WMH load and cognitive function varies by suicide attempt status.

**Method:**

This cross-sectional study included 58 adults aged 65 years and older. Participants were categorized into three groups: RSA (*n* = 17), NS (*n* = 20), and HC (*n* = 21). All participants underwent brain MRI and completed multiple psychological evaluations and neurocognitive tests, including the Cognitive Abilities Screening Instrument. We compared the three groups with respect to cognitive function, WMHs, and the relationship between WMH load and cognitive performance.

**Results:**

LLD patients with a recent suicide attempt (RSA) had significantly poorer global cognitive function and greater periventricular WMH compared to non-suicidal LLD patients and healthy controls. Greater WMH was significantly associated with lower cognitive function only in the suicide-attempt group.

**Conclusion:**

Worse global cognitive function and greater WMHs may collectively increase the risk of suicidal attempts in LLD patients.

## Introduction

Suicide constitutes a major global health problem. Suicide rates among older adults are higher than among young adults in all countries, contributing greatly to global burden of disease [1]. Investigating the risk factors and neural mechanisms of late-life suicide is crucial, particularly because suicide in older adults is characterized by high suicidal intent and lethality [2]. Risk factors for suicide in older adults include depression, physical illness, pain, and functional impairment [3]. With regard to mental illnesses, depressive disorder is most strongly associated with suicide among older adults [4]. Thus, investigating the etiology of late-life depression (LLD) may provide insight into suicidality among older adults.

A growing number of studies have suggested biological causes of LLD. The vascular depression hypothesis, a central framework in the pathophysiology of LLD, posits that cerebrovascular illness may induce or prolong depression in older adults [5]. In addition, this hypothesis emphasizes the crucial role of white matter hyperintensities (WMHs), which are strongly associated with cerebrovascular risk factors and may interrupt the neural circuits regulating emotion and cognition [6]. Experts estimate that the prevalence of depression among community-dwelling older adults is approximately 13.5% [7]. However, most individuals with LLD have never attempted suicide; this implies that LLD patients with a recent suicide attempt (RSA) may belong to a unique subgroup.

Growing evidence suggests that neurobiological factors may partially mediate the predisposition to suicide. Studies have identified WMHs as a potential biomarker for suicide in young and middle-aged adults [8]. The association between WMH and cognitive impairment in older adults is well-established [9]; if WMH is also associated with suicide in older adults, differences in cognition may exist between LLD patients who do versus do not attempt suicide. However, most existing literature focuses on the relationship between WMH, cognitive function, and LLD. Knowledge of WMH and cognitive function–associated suicidality in LLD patients remains limited. Few studies have investigated the association between suicide in older adults and WMH and cognitive function. In fact, a literature search using the four keywords: WMHs, cognition, aged and suicide, attempted yields only one relevant study [10], in which Lin et al. reported that suicide attempters with late-life depression had greater periventricular WMH and poorer executive function, possibly reflecting an underlying cerebrovascular mechanism of cognitive vulnerability.

This study aims to examine differences in cognitive performance and WMH burden across three clinical groups: LLD patients with recent suicide attempts (RSA), non-suicidal LLD patients (NS), and healthy controls (HC). We further investigate whether the relationship between WMH load and cognitive function differs among these groups. We hypothesize that LLD patients with recent suicide attempts will exhibit both poorer cognitive performance and greater WMH burden compared to the other two groups. Furthermore, we anticipate that NS patients will show intermediate levels of cognitive impairment and WMH burden—greater than HC but less severe than RSA—reflecting a gradation of severity across the three groups. Moreover, we expect that increases in WMH burden will be more strongly associated with cognitive decline in the RSA group than in the NS and HC groups, indicating a potential neurobiological vulnerability reflected by a significant WMH-by-group interaction.

## Methods

### Participants and study design

This is a cross-sectional study with observational study design. Being a exploratory study, it included 58 adults aged 65 years or older (mean age = 74.6 years) and three types of participants were recruited.

Group 1 (described as RSA) included patients recruited from a psychiatric inpatient unit who had recently engaged in self-injurious behavior with the intent to die; these patients met the *DSM-5* criteria for major depressive episode, and diagnoses were confirmed by a board-certified geriatric psychiatrist and a board-certified neurologist (Y.T. Lee and L.K. Huang, respectively). The RSA group was referred to by the researcher’s hospital’s Suicide Prevention Center. Per Taiwan’s suicide prevention policy, cases must be reported within 24 h of awareness, leading to immediate care and referral, with most RSA cases included within one week of self-injurious behavior.

Group 2 (described as non-suicidal/NS) comprised patients recruited from psychiatric outpatient facilities with major depression and no history of self-harm behavior, idea of death, suicidal ideation, or suicide attempts. The group included patients who met the criteria for a DSM-5 diagnosis of nonpsychotic unipolar major depressive episode.

Group 3 (described as healthy controls/HC) comprised older adults with no history of major psychiatric disorder, suicidal ideation, or suicide attempt. Those with major neurological illnesses were excluded. The HC group was primarily recruited through advertisements.

With respect to other inclusion and exclusion criteria, the study only included patients with no remarkable suicide sequelae. None had attempted suicide-related head injuries. This study also excluded patients who may have experienced anoxic brain injuries resulting in clinical cognitive decline, physically unstable patients, those with a history of major psychiatric disorders other than depression, and those with cognitive disorders, major neurological illnesses, and brain injuries.

The Cognitive Abilities Screening Instrument (CASI) [11] was implemented as a global measure of cognitive function. Additionally, to exclude the comorbidity of cognitive disorders, Mini-Mental State Examination (MMSE) scores [12] were estimated from subsets of CASI items. All participants were required to meet the CASI-derived MMSE cutoff scores of 24 or above (for participants with more than 6 years of schooling), 21 or above (for participants with 1 to 6 years of schooling), and 17 or above (for participants with no schooling). MMSE cutoff scores were not implemented for patients aged 85 years and older; these patients were simply required to complete the neuropsychological tests and assessments [13]. Some patients with severe depression had MMSE scores below the cutoff points. These patients were followed for 8 weeks of treatment and were enrolled in the study if their MMSE scores improved during that time [14].

The authors assert that all procedures contributing to this work comply with the ethical standards of the relevant national and institutional committees on human experimentation and with the Helsinki Declaration of 1975, as revised in 2008. All procedures involving human subjects/patients were approved by the Ethics Committee of the Taipei Medical University Joint Institutional Review Board (IRB: N201707019). Methods followed guidelines and regulations and all participants provided written informed consent.

### Assessments

#### Clinical assessments

The 15-item geriatric depression scale (GDS-15) evaluated depression severity; a score of 6 or above indicated LLD [15]. Because impulsivity is also linked with suicide [16], we employed the Barratt Impulsiveness Scale version 11 (BIS-11) [17]. The Patient Health Questionnaire-15 (PHQ-15) [18] and the Whiteley Index (Whiteley-7) [19] assessed somatization and health anxiety, respectively. The Charlson Comorbidity Index questionnaire (CCIQ) [20] and 10-point Numerical Rating Scale (NRS) [21] evaluated comorbidity and pain respectively.

#### Cognitive assessments

All participants completed the CASI [11] to assess neurocognitive function. CASI is one of the few cognitive screening tools that have been validated for use in even illiterate or low-literate older adults [22]. The total score ranges from 0 to 100, with higher scores indicating better cognitive performance. The reference cutoff scores for CASI are as follows: illiterate individuals (0 years of schooling, unable to read or write): 49/50; partially educated individuals (1–5 years of schooling, partially literate): 67/68; educated individuals (≥ 6 years of schooling, fully literate): 79/80. The CASI assesses cognitive functioning in nine domains: long-term memory, short-term memory, attention, mental manipulation, orientation, abstract thinking, language, drawing, and verbal fluency. The total score constitutes an objective measure of global cognitive function. In addition, MMSE scores [12] were estimated from subsets of the CASI items and the total score ranges from 0 to 30, with higher scores indicating better cognitive performance. In this exploratory study, the MMSE total score was used as an inclusion/exclusion criterion to exclude the comorbidity of cognitive disorders.

### Procedures

The study was conducted in a psychogeriatric inpatient ward and in the outpatient department of a university-affiliated hospital. Participants were evaluated within 4 weeks of psychiatric admission or at the beginning of 4 weeks of outpatient treatment. Psychological and neurocognitive assessments were completed separately by a geriatric psychiatrist and a psychologist, within a time period of 2–2.5 h. Routine clinical care continued to be administered during the evaluation.

### MRI acquisition

MRI was conducted with a 3 T unit (GE MR750) in a teaching hospital. T2-weighted-fluid-attenuated inversion recovery MRI (FLAIR MRI) sequence with a slice thickness of 3–5 mm was employed to evaluate WMH. The Fazekas scale was used to rate the degree of WMH severity based on the axial FLAIR images. Periventricular and deep WMH were assessed separately. Periventricular hyperintensities were graded as absent (grade 0), cap (grade 1), smooth halo (grade 2), or irregular and extending into the subcortical white matter (grade 3); deep WMH was graded as absent (grade 0), punctate foci (grade 1), early confluent (grade 2), or confluent (grade 3) [23].

### Statistical analysis

Descriptive statistics characterize mean and standard deviation for quantitative variables, counts and range of variation for qualitative variables. Normal distribution of quantitative variables was visually inspected using the graphical method since normality tests suffer from little power for small sample sizes [24]. Parametric (normal distribution) or non-parametric inferential statistical methods were conducted accordingly. Chi-square or F-test were employed to summarize demographic data differences between groups. The Bonferroni correction was applied in post-hoc tests. Analysis of covariance (ANCOVA, adjusted for sex, age, and depression severity) was implemented to compare the WMH load. Based on previously published literature [10], generalized linear models (GLMs) were used to analyze data across the three groups. The dependent variables included the CASI total score and its subdomain scores. Covariates such as sex, age, years of schooling, and GDS-15 scores were controlled for. Additionally, independent variables of WMH load (total, periventricular or deep) and suicide attempt groups were examined. To examine potential interaction effects, an interaction term between group and WMH was incorporated into the model. Finally, educational background was excluded for two reasons: its correlation with GDS-15 (*r* = 0.22) raised multicollinearity concerns, potentially affecting model stability and interpretability. Additionally, with a limited sample size, we prioritized covariates that maximize explanatory power while maintaining statistical power, as including education risked overfitting and reducing model robustness. Table 1; Fig. 2 present results derived from a single GLM, in which total WMH load was entered as the independent variable and the CASI total score as the outcome variable. Non-standardized regression coefficients (β) with 95% confidence intervals (CIs) were reported. A statistically significant interaction term indicated that the correlation between group and WMH in the GLM model was dependent on the group. All analysis was performed with 3 group classifications according to the afore mentioned principles. Given the small sample size, effect sizes were assessed using partial eta squared for all covariates and independent variables. Data were analyzed with SAS v9.2.

**Table 1:** Demographic and clinical characteristics of older adults with a recent suicide attempt, non-suicidal late-life depression and healthy control

	HC (*n* = 21)	NS (*n* = 20)	RSA (*n* = 17)	F or ꭓ^2^	*p*	Post-hoc
Age, years	74.00 (6.59)	74.35 (6.47)	75.53 (8.44)	0.23	0.795	
Men (%)	4 (19.0)	2 (10.0)	3 (17.6)	0.72	0.697	
Education, years	7.38 (4.74)	5.65 (3.88)	5.82 (4.65)	0.94	0.399	
*Psychological and Physiological Scales*						
GDS-15	2.05 (1.50)	9.20 (3.29)	10.88 (3.02)	60.25	< 0.001^***^	HC < NS, RSA
PHQ-15	3.71 (3.29)	7.00 (6.27)	5.94 (3.09)	2.84	0.067	
Whiteley-7 Scale	5.48 (1.75)	5.05 (2.33)	5.00 (2.45)	0.29	0.751	
BIS-11	59.05 (7.39)	61.80 (5.90)	57.88 (5.88)	1.83	0.171	
CCI	0.19 (0.51)	0.45 (0.83)	0.41 (0.62)	0.90	0.411	
NRS Numerical Rating Scale	0.33 (0.91)	1.45 (2.52)	1.00 (2.00)	1.77	0.180	
*Neurocognitive function*						
CASI						
Total score	83.05 (12.63)	75.90 (11.32)	67.59 (19.84)	5.18	0.009^**^	HC > RSA
Long term memory	9.52 (0.81)	9.00 (1.78)	8.82 (1.70)	1.19	0.311	
Short term memory	9.24 (3.05)	9.10 (2.69)	7.29 (4.03)	2.01	0.144	
Attention	7.00 (1.61)	6.05 (1.00)	5.29 (2.08)	5.44	0.007^**^	HC > RSA
Mental manipulation	7.52 (2.60)	7.30 (3.84)	5.94 (3.29)	1.24	0.296	
Orientation	16.24 (2.39)	13.95 (3.50)	11.59 (5.03)	7.46	0.001^**^	HC > RSA
Abstract thinking	8.38 (1.96)	8.35 (1.95)	7.76 (2.44)	0.49	0.616	
Language	9.00 (1.48)	8.55 (1.99)	8.47 (2.07)	0.47	0.625	
Drawing	9.57 (0.98)	8.40 (2.72)	7.71 (3.20)	2.91	0.063	
Verbal fluency	6.67 (2.50)	5.75 (2.29)	4.88 (1.54)	3.16	0.050	
*WMH*						
Total WMH	1.85 (1.46)	1.45 (1.85)	2.88 (2.00)	3.15	0.051	
Periventricular WMH	0.85 (0.75)	0.65 (0.93)	1.59 (1.28)	4.46	0.016^*^	RSA > NS
Deep WMH	1.00 (1.03)	0.80 (1.01)	1.29 (1.05)	1.07	0.349	

*HC*, Healthy control; *NS*, Non-suicidal late-life depression; *RSA*, Late-life depression with a Recent suicide attempt; *GDS-15*, 15-Item Geriatric Depression Scale; *PHQ-15*, Patient Health Questionnaire-15; *Whiteley-7 Scale*, Whiteley-7 Index Questionnaire; *BIS-11*, Barratt Impulsiveness Scale–11; *CCI*, Charlson comorbidity index score; *NRS*, Numerical Rating Scale; *CASI*, Cognitive Abilities Screening Instrument; *WMH*, White matter hyperintensities

*p < 0.05, **p < 0.01, ***p < 0.001

**Fig. 1:**
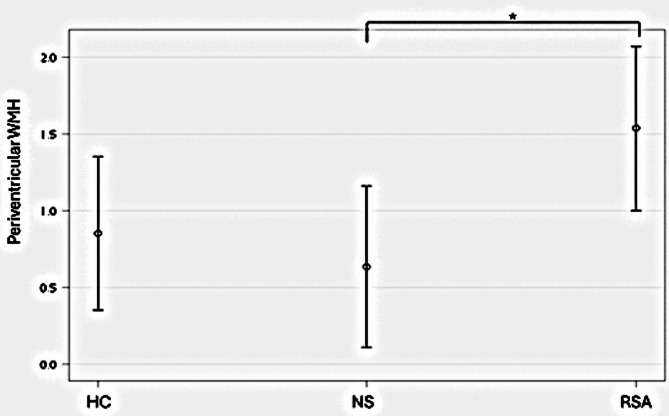
Differences in periventricular WMHs (adjusted for age and sex;Mean of WMHs; Error bars: 95% CI). *HC*, Healthy control; *NS*, Non-suicidal late-life depression; *RSA*, Late-life depression with a recent suicide attempt; *WMHs*, white matter hyper-intensities; *CASI*, Cognitive Abilities Screening Instrument; *GDS-15*, 15-Item Geriatric Depression Scale. *p < 0.05, Bonferroni corrected

**Fig. 2:**
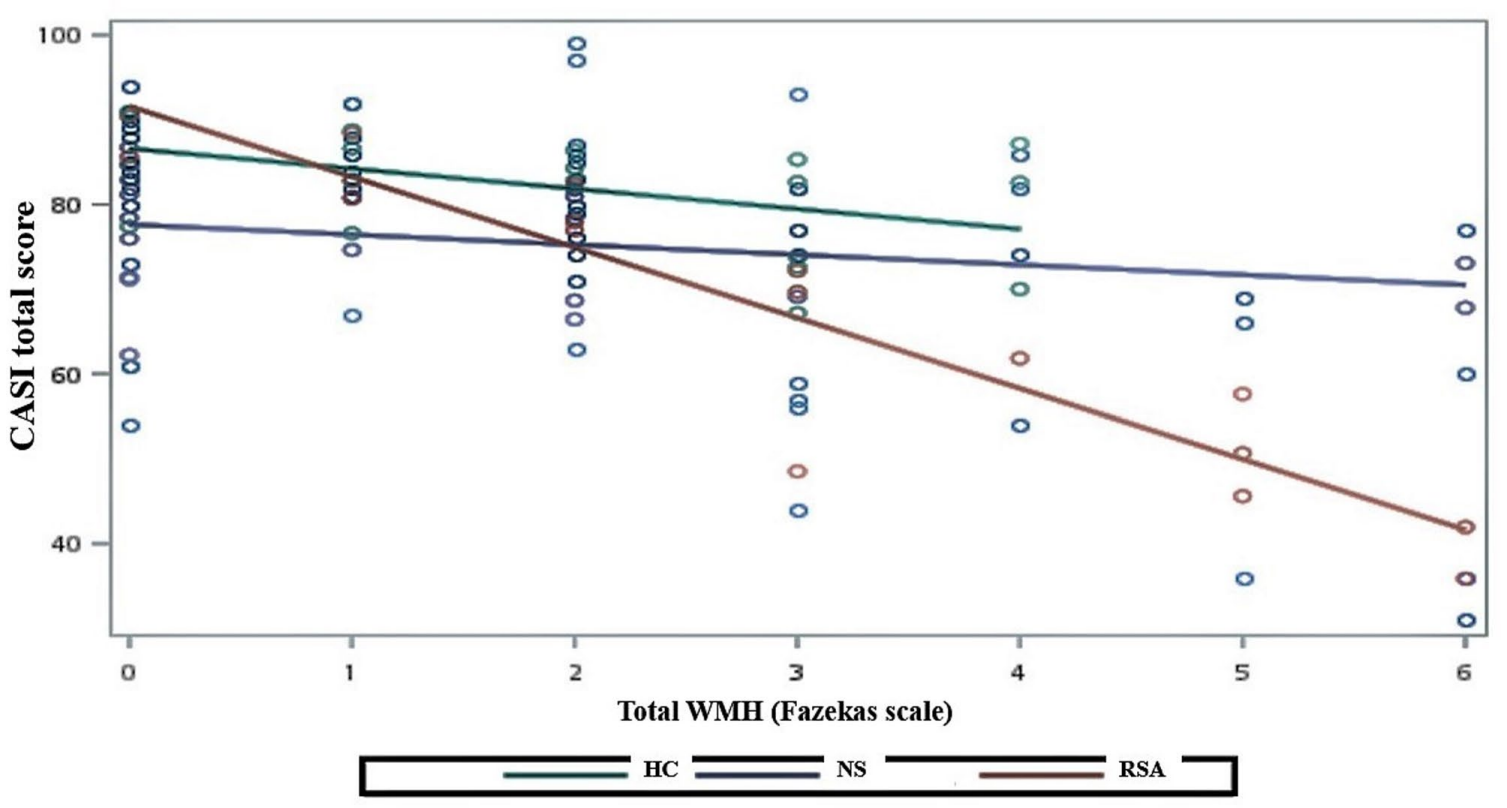
Interaction effect between WMH load and group status on CASI total scores (adjusted for age, sex, and GDS-15). *HC*, Healthy control; *NS*, Non-suicidal late-life depression; *RSA*, Late-lifedepression with a recent suicide attempt; *WMHs*, white matter hyperintensities;*CASI*, Cognitive Abilities Screening Instrument; *GDS-15*, 15-ItemGeriatric Depression Scale

## Results

The study enrolled a total of 58 participants divided into 3 groups, HC (*n* = 21; average age = 74.0 ± 6.59 years), NS (*n* = 20; average age = 74.35 ± 6.47 years), and RSA (*n* = 17; average age = 75.53 ± 8.44 years). Table 2 compares the demographic characteristics of the three groups, including age, sex, and education. All groups were predominantly female. Although the NS and RSA groups had lower average education levels compared to the HC group, the differences were statistically non-significant (Table 2).

The patients in NS and RSA groups shared a similar severity of depression and had significantly higher GDS-15 scores compared to those in HC. All other psychological and physiological variables did not show statistically significant differences among the three groups (Table 2).

**Table 2:** Generalized linear model of CASI total scores in relation to total WMH and group interaction

	β	95% CI	*p*-value	Partial eta2
Intercept	156.246	123.777, 188.716	< 0.001	
Age	− 0.911	−1.359, −0.464	< 0.001***	0.259
Sex	−5.358	−12.766, 2.050	0.152	0.042
GDS-15	− 0.282	−1.360, 0.795	0.601	0.006
WMH	− 0.793	−4.065, 2.478	0.628	0.127
*Group* (HC as reference)				0.058
RSA	4.243	−9.598, 18.085	0.540	
NS	−4.641	−16.532, 7.248	0.436	
*Group x WMH* (HC as reference)				0.177
RSA	−4.948	−9.024, −0.872	0.018*	
NS	0.452	−3.637, 4.542	0.825	

*HC*, Healthy Control; *NS*, Non-suicidal late-life depression; *RSA*, Late-life depression with a recent suicide attempt; *WMHs*, white matter hyper-intensities; *CASI*, Cognitive Abilities Screening Instrument; *GDS-15*, 15-Item Geriatric Depression Scale

*p < 0.05, **p < 0.01, ***p < 0.001

With respect to cognitive status, significant group differences were observed in the total CASI scores, with RSA performing worse than HC. Additionally, group differences were found in two cognitive subdomains—CASI Attention and CASI Orientation—where RSA also scored lower than HC. Patients in RSA generally scored significantly lower compared to HC in global cognitive function, attention, and orientation (Table 2).

Regarding WMH load, only periventricular WMH showed a statistically significant difference among the three groups, with RSA exhibiting a significantly higher WMH load than NS (Table 2). Additionally, after adjusting for age, sex, and GDS-15 score, the results remained consistent, with post-hoc test results for WMH revealed significantly greater periventricular WMH in the RSA group compared to the NS group (*p* = 0.04, Bonferroni corrected, eta squared = 0.1183; Fig. 1).

In the GLM analysis, after adjusting for age, sex, and GDS-15 score, a significant interaction effect was observed only in the RSA group between total WMH and total CASI score (ß = − 4.948, 95% CI −9.024 to −0.872, *p* = 0.018; Table 1). Furthermore, only within the RSA group, was higher total WMH significantly associated with lower total CASI scores (Fig. 2).

## Discussion

The major observations of this study are as follows: First, patients with late-life depression (LLD) and a recent suicide attempt (RSA) demonstrated lower global cognitive function, particularly in attention and orientation domains, compared to healthy controls (HC). Second, these patients exhibited a greater white matter hyperintensity (WMH) burden, especially in the periventricular region, than both non-suicidal LLD patients and HC. Third, among patients in the RSA group, higher WMH load was significantly associated with lower global cognitive scores, a relationship not observed in the non-suicidal LLD or HC groups. Overall, these findings are consistent with the hypotheses presented in the Introduction. This study contributes to the limited literature examining whether global cognitive impairment, WMH load, and their association differ according to suicide attempt status among individuals with LLD. Given the exploratory nature of our analysis and reliance on general cognitive screening measures (CASI), these findings should be interpreted with caution. Further studies incorporating comprehensive neuropsychological assessments are warranted.

While some research suggests that risk factors for late-life suicide may contribute to suicidal behavior in only a subset of individuals, the nature underlying suicidal diathesis in older adults remains poorly understood [25]. WMH is common in aging populations and has been associated with both depression and cognitive decline. However, its role in suicide risk among LLD patients remains an area requiring further investigation.

Our findings suggest that LLD patients with an RSA demonstrated lower cognitive performance in global cognitive functions, attention, and orientation compared to the HC group. Previous studies have indicated that various cognitive domains, including executive functioning, attention, processing speed, and visuospatial abilities, may be affected in LLD [26]. Some research has reported that individuals with LLD and a history of suicide attempts tend to exhibit lower executive function scores [25, 27]. Lin et al. found that LLD patients with a history of suicide performed worse on the Digits Forward Test and the Letter–Number Sequencing task compared to both non-suicidal LLD patients and HC participants [10]. Given that LLD patients with RSAs may represent a specific subgroup within LLD, our study suggests that this group may have poorer cognitive function in multiple domains, including global cognitive function. Similar trends have been reported by Dombrovski et al., who found that older adults with LLD and a history of suicide attempts had lower scores in executive function, global cognitive function, and attention and memory subscales [27]. However, Lin et al. [10] reported no significant difference in global cognitive function (measured by MMSE total score) between LLD patients with a history of suicide attempts and the control group. This discrepancy may be due to two factors: (1) CASI provides a more detailed and comprehensive assessment of cognitive function compared to MMSE, and (2) our study applied stricter inclusion criteria, focusing on individuals aged 65 and older with a recent, well-documented suicide attempt. Given the exploratory nature of our findings and the use of cognitive screening tools, further neuropsychological evaluation is needed to confirm these results. More broadly, emerging evidence suggests that cognitive difficulties may be a risk factor for suicide attempts, possibly due to impairments in problem-solving and adaptability. Older adults experiencing cognitive decline may have a reduced capacity to cope with life stressors, and suicidal behavior may emerge as a maladaptive coping mechanism in response to perceived losses or physical health challenges [28, 29].

Our findings also suggest that LLD patients with an RSA had a higher WMH load, particularly in the periventricular area. Some studies in younger and middle-aged populations have proposed that WMH burden may serve as a potential biomarker for suicidality [8]. A meta-analysis reported that individuals with a history of suicide attempts had significantly greater WMH burden than those without [30]. Additionally, a study examining WMH load in LLD patients with a history of suicide suggested that prior suicide attempts were associated with higher WMH burden [31]. Another study found that in older adults with suicide attempts, periventricular WMH was more strongly associated with cognitive function and processing speed than deep WMH [32]. These patterns align with our observation that LLD patients with an RSA had greater periventricular WMH burden and poorer global cognitive function. Importantly, WMH burden has been linked to poorer cognitive function in LLD [33], and it is hypothesized that WMH may compromise cognitive flexibility and stress adaptation, increasing vulnerability to suicidal behavior [31]. The observed higher WMH burden in LLD patients with an RSA may further impair cognitive coping mechanisms and contribute to suicide risk.

Neurocognitive dysfunction has been proposed as a factor that may trigger suicidal crises under stressful conditions [34]. The “disconnection hypothesis” in LLD suggests that WMH may disrupt both emotion regulation and executive function, contributing to suicide risk [6, 35]. Specifically, periventricular and deep WMH may impair long association fibers that connect cortical and subcortical regions, as well as short U-fibers connecting neighboring cortical areas [36]. This neurobiological model supports the hypothesis that greater periventricular WMH burden in older adults with LLD and an RSA may interfere with neural circuits critical to regulating emotion, cognition, and behavior, potentially contributing to executive function deficits [25] and social-emotional dysfunction [37].

Our exploratory results suggest a tendency for greater WMH load to be associated with lower global cognitive function, specifically in LLD patients with an RSA. A meta-analysis has reported that WMH is significantly associated with poorer cognitive performance across all examined domains, including global cognition [38]. Regarding cognitive deficits in LLD, previous studies have suggested a potential link between WMH and cognitive dysfunction, including global cognition [39]. Additionally, Kramer-Ginsberg et al. observed an interaction between WMH severity and global cognitive function across various domains in individuals diagnosed with LLD [40]. Like our findings, Lin et al. reported that a higher WMH load was associated with lower cognitive performance, particularly in LLD patients with a history of suicide attempts [10]. This may indicate that the potential impact of WMH on cognition is more pronounced in this subgroup of LLD patients. However, given the exploratory nature of this study, the small sample size, and the use of cognitive screening measures, these findings should be interpreted thoughtfully. Further research with larger samples and comprehensive neuropsychological assessments is necessary to validate these observations.

Krishnan et al. proposed the theory of *MRI-defined vascular depression*, which includes MRI findings indicative of cerebrovascular disease [41]. Some research examining this theory suggests that vascular depression may represent a distinct subtype of LLD, potentially characterized by specific neuropsychological features such as executive dysfunction and reduced processing speed [42, 43]. Additionally, studies have indicated that WMH may play a role in vascular depression [6, 42]. Given the established association between WMH and poorer cognitive function [38], it is possible that this concept may help contextualize our exploratory finding that LLD patients with an RSA exhibit greater WMH burden and are a subtype of LLD. However, considering the small sample size and the use of cognitive screening measures in this study, further research is required to clarify this finding.

Our study has several noteworthy strengths and some limitations. Firstly, previous studies on LLD patients with suicide attempts have often included both recent and historical attempts, leading to a broad range of participants. In contrast, the current study exclusively included LLD patients who have recently attempted suicide, allowing for a more targeted examination of suicidal behavior in older adults. Secondly, the study has implemented a strict age cutoff for older adults and has an average patient age of approximately 75 years, which increases the clinical relevance of the findings by focusing on a real population of older adults. Additionally, the study has a flexible standard of MMSE based on years of schooling and ensured that all LLD participants over 80 years of age were followed for at least 3 months to confirm the exclusion of dementia. Lastly, all participants with recent suicide attempts were inpatients, which allowed for a more stable physical and psychological state during the administration of neurocognitive tests.

Regarding the study’s limitations, a key limitation of this study is the lack of control for educational attainment, which is known to significantly influence cognitive performance in older adults. This omission may confound the associations observed in our findings—particularly the relationship between WMH burden and global cognitive function. Specifically, individuals with lower educational attainment may exhibit reduced CASI scores independent of neurobiological pathology, while those with higher educational backgrounds may demonstrate cognitive resilience despite comparable WMH burden. This could lead to an underestimation or overestimation of the true strength of the association between WMHs and cognitive performance. Education was excluded not by oversight but due to limited sample size and Taiwan’s unique sociopolitical history. In fact, nearly 30% of the SA group in our study received education entirely in Japanese under colonial rule, only to later live in a society where Japanese was banned. This linguistic and cultural rupture renders standard measures of “years of education” unreliable. In this context, adjusting for education may have introduced more bias than clarity. Future studies with larger samples should address this limitation by incorporating culturally appropriate educational controls to better understand the interplay between WMHs, cognition, and suicidality in late-life depression. Other limitations include its exploratory nature with small sample size and reliance on cognitive screening measures are key factors that must be acknowledged. These limitations warrant cautious interpretation of the findings. The statistical power ranged from 0.11 to 0.95 for the CASI total score and its subdomains, some non-significant results may be attributed to low statistical power, highlighting the need for future studies with larger sample sizes. Despite the limited sample size, sufficient statistical power was observed for comparisons involving the CASI total score, Attention, and Orientation. Nonetheless, a larger sample size will be necessary in future studies to enhance the generalizability of our findings. The limitation of the small sample size was acknowledged explicitly, and the necessity of future studies involving larger participants and in-depth neuropsychological assessments was emphasized. Besides, the assessment of WMH load was conducted using a visual rating method. The literature presents conflicting views on the distinction between periventricular and deep WMH, with some suggesting a high correlation between them and others emphasizing the distinctions based on etiological evidence and quantitative MRI analysis [30, 44, 45]. Further research utilizing various imaging modalities is necessary to investigate this ongoing debate. Additionally, our study had more women than men, which may be due to LLD differences or cultural norms. Older men in Asia often avoid psychiatric help or research on suicide due to stigma [46]. However, older men tend to use more lethal methods in suicide attempts [47], making studying this group crucial. The inclusion criteria yielded a relatively small proportion of older adults with recent suicide attempts, limiting the statistical power and possibly reducing generalizability. Lastly, the study did not take into account certain characteristics such as the age of the first onset of depression and the number of depressive episodes which were not available in the oldest-old in our sample. This limitation is particularly significant and must be addressed in future research.

## Conclusion

This exploratory study suggests that LLD patients with an RSA exhibited lower cognitive screening performance and a greater periventricular WMH load. Additionally, older adults with LLD and an RSA may have a biological susceptibility to cognitive difficulties associated with WMH. The combination of lower cognitive screening scores and greater WMH burden may contribute to an increased risk of suicide attempts in LLD patients. However, given the small sample size and the use of cognitive screening measures rather than comprehensive neuropsychological assessments, these findings should be interpreted carefully. Further studies with larger samples and more detailed cognitive evaluations are needed to confirm these observations.

## Data Availability

No datasets were generated or analysed during the current study.

## Abbreviations

LLD: Late-life depression
HC: Healthy control
NS: Non-suicidal late-life depression
RSA: Late-life depression with recent suicide attempts
GDS-15: 15-item geriatric depression scale
CASI: Cognitive abilities screening instrument
WMH: White matter hyperintensities
